# Noyes-Whitney Dissolution Model-Based pH-Sensitive Slow Release of Paclitaxel (Taxol) from Human Hair-Derived Keratin Microparticle Carriers

**DOI:** 10.1155/2021/6657482

**Published:** 2021-05-07

**Authors:** V. W. Wimalasiri, S. P. Dunuweera, A. N. Dunuweera, R. M. G. Rajapakse

**Affiliations:** ^1^Department of Chemistry, Faculty of Science, University of Peradeniya, Sri Lanka; ^2^Postgraduate Institute of Science, University of Peradeniya, Sri Lanka; ^3^Department of Basic Science, Faculty of Allied Health Sciences, University of Peradeniya, Peradeniya 20400, Sri Lanka

## Abstract

This paper describes a convenient and straightforward method developed to extract keratin particles (KPs) from human hair. It also involves their characterization by several methods and encapsulation of the anticancer drug Paclitaxel (Taxol) within them, aiming for targeted delivery to cancerous sites and slow release at their vicinity. The KPs obtained were in micrometer in size. They are capable of encapsulating Taxol within them with a high encapsulation efficiency of 56% and a drug loading capacity of 2.360 g of Taxol per g keratin. As revealed by the SEM elemental analysis, KPs do not contain any toxic metal ion, and hence, they pose no toxicity to human cells. The pH-dependent release kinetics of the drug from KPs indicates that the drug is released faster when the pH of the solution is increased in the 5.0 to 7.0 pH range. The release kinetics obtained is impressive, and once targeted to the cancerous sites, using cancer directing agents, such as folic acid; a glutamate urea ligand known as DUPA; aminopeptidase N, also known as CD13; and FAP-*α*-targeting agents, the slow release of the drug is expected to destroy only the cancerous cells. The Noyes-Whitney dissolution model was used to analyze the release behavior of Taxol from KPs, which shows excellent fitting with experimental data. The pH dependence of drug release from keratin is also explained using the 3-D structures and keratin stability at different pH values.

## 1. Introduction

Cancer is a group of diseases that primarily occurs due to uncontrollable cell growth where the cells that are supposed to die would survive while forming new cells. These extra cells can further divide rapidly in an uncontrollable manner to form growths known as tumors [1]. Secondaries result when a tumor successfully spreads to other parts of the body and grows difficult to treat [2]. Cancers inherited from parents are usually due to genetic reasons. Damage to deoxyribonucleic acid (DNA) due to certain environmental exposures could lead to cell division errors. These exposures can be radiation (ultraviolet radiation from the Sun), hazardous chemicals such as those in tobacco smoke, etc. [3]. Cancer treatment is done in three major ways: surgery, radiation therapy, and chemotherapy. Surgery can remove isolated cancers, and radiation therapy is also useful in killing isolated cancers, while chemotherapy could work throughout the body. There are three main goals of chemotherapy, which are a cure, control, and palliation. Certain cancers can be cured with some therapeutic drugs, particularly at the early stage of cancer, before it spreads into other cells/organs. However, cancers could be controlled and managed, with the help of chemotherapeutic drugs, as a chronic disease, even when it is incurable [4]. When they have spread into other parts of the body at the later stages of cancers, cure or control may not be possible. In such circumstances, chemotherapy is used to ease the symptoms caused by cancer. This is known as palliation. Chemotherapy could be the only treatment needed or used in conjunction with surgery and/or radiation therapy. Some other advantages of chemotherapy are that it could be used to shrink cancer to assist in an effective surgery or radiation therapy (neoadjuvant therapy), it could be used after surgery or radiation therapy to destroy any remaining cancer cells (adjuvant therapy), and it may be used with other treatments [5].

Chemotherapeutic drugs can also adversely affect healthy cells causing side effects. It has been reported that normal cells most likely affected by chemotherapeutic drugs are blood-forming cells in the bone marrow, hair follicles, and cells in the mouth, digestive tract, and reproductive system [6]. Some chemotherapeutic drugs can damage cells in the heart, kidneys, bladder, lungs, and nervous system also [7]. Conventional drug administration techniques are not suitable for cancer treatments since these are not targeted techniques. Hence, the drug is available throughout the body adding risks to the above side effects. The conventional drug delivery systems have also been proven to contain many limitations such as low accurate targeting, low therapeutic indices, poor water solubility, and drug resistance induction [8]. A new concept of nanoparticle-based drug delivery systems has been introduced to overcome these drawbacks, in which nanoparticles are used as drug carriers. This is a Targeted Drug Delivery System (TDDS) where a drug is released at a preselected biosite in a controllable manner [8]. Nanoparticle-based drug carriers can enhance the therapeutic effectiveness of the drugs, which are carried by practicing active targeting for the site-specific delivery and passive targeting such as Enhanced Permeability and Retention (EPR) by making them water-soluble to improve the suboptimal pharmacokinetics in limited water-soluble delivery methods [9]. These types of drug delivery systems have been designed to insert a therapeutic agent in the required amount, at the right time, to the proper location in the body so that it optimizes efficiency, increases compliance, and minimizes side effects [10]. There are several different types of nanocarriers that are used to encapsulate, covalently bind, or entrap drugs. These include liposomes [11], dendrimers [12], hydrogels [13–15], polymer-drug conjugates [16], inorganic nanoparticles [17–20], lipid-polymer hybrid nanoparticles [21], magnetic nanoparticles [22], and quantum dots [23]. However, natural materials offer several distinct advantages, such as specialized cell-cell communications, which synthetic carriers may not have. The use of nucleic acids/peptides [24] and exomes [25] as drug carriers has been investigated. With the development of DNA technology, nucleic acids such as DNA have been used in constructing artificially designed nanostructures, which are used in TDD based upon directly sensing their environments. Nucleic acid logic circuits have been developed to be used as the core of a system that could release a drug only in response to a specific mRNA stimulus [26]. Nanoscale folding of DNA to create nonarbitrary two- and three-dimensional shapes at the nanoscale, known as the DNA origami method, has been used to design a DNA box with a controllable lid for a drug to be encapsulated within the box. The lid is to be opened only in response to the desired stimulus [27].

This study used keratin nano- and microparticles extracted from human hair to encapsulate the anticancer drug Paclitaxel (Taxol) for targeted delivery and slow release. Previous studies show that Taxol has been encapsulated in polymer nanocarriers such as poly(lactic-co-glycolic acid) (PLGA) [28], chitosan-modified PTX-loaded PLGA nanoparticles [29], didodecyl dimethylammonium bromide- (DDAB-) stabilized PLGA nanoparticles [30], bare and modified carbon nanomaterials [31], bare and modified magnetic nanocarriers such as Fe_3_O_4_ [32] and gold [33], and many other nanocarriers such as micelles [34]. A brief write-up of Taxol, keratin, and their structures is given in Supplementary Materials (S1, S2, and Fig. S2). Keratin has the advantage that it is available naturally, mostly in hair, and it can be isolated in nano-microsized particles quite easily, as shown here. This study constitutes the extraction, purification, characterization, and TDD applications for Taxol of keratin nano-to-microparticles.

## 2. Materials and Methods

All the chemicals, except Taxol, were of analytical grade, which was purchased from Sigma-Aldrich and was used without further purification. Taxol injection bottles containing 6 mg Paclitaxel U.S.P. in Polyoxyl 35, 527 mg of Castor Oil U.S.N.F., and 49.7% *v*/*v* dehydrated alcohol were purchased from Sri Lanka Pharmacy, Kandy, Sri Lanka, and Raj Pharmacy, Kandy, Sri Lanka. Human hair was collected from the University of Peradeniya Barbershop, which was of students of age in the midtwenties and perfect black color. Water used was distilled and deionized water obtained from Milli-Q.

### 2.1. Preparation of Keratin Particles

10.0 g of human hair sample was immersed in absolute ethanol (100% EtOH) solution for 24 h with stirring. The hair sample was then washed several times, with distilled water several times, and dried for 6 h at 50°C. Solution A (0.50 mol dm^−3^ NaOH(aq) solution) and Solution B (0.50 mol dm^−3^ NaHSO_3_(aq) solution) were prepared. 10.0 g of hair sample was added into a 400 mL beaker, and 250.0 mL of Solution A and 16.60 mL of Solution B were added to obtain a 15 : 1 molar ratio of OH^−^(aq) : HSO_3_^−^(aq) in the solution. The mixture was heated up to 80°C, and the temperature was maintained at this value for 2 h while stirring at 200 rpm. The solution was then allowed to cool down to room temperature gradually. It was then filtered using a Buchner funnel, and the filtrate was treated with 35.5% *v*/*v* HCl solution until a precipitate was obtained. The precipitate was filtered using the Buchner funnel, washed with distilled water, and dried at 50°C for 2 h [35]. The material thus obtained was characterized by X-ray Diffraction (XRD) (Siemens D5000 X-ray Powder Diffractometer, 5°–80° range with a time step of 0.5°), environmental scanning electron microscopy (SEM) with EDAX Facilities (Hitachi SU6600), transmission electron microscopy (TEM) (JEOL JEM-3010 TEM operating at 300 kV), laser light scattering-based particle size analysis (CILAS Particle Size Analyzer NANO DS), and Fourier transform infrared (FT-IR) spectroscopy (Shimadzu IR Prestige-21 spectrometer using the KBr pellet).

### 2.2. Determination of Encapsulation Efficiency of Taxol

1000 ppm Taxol solution was prepared by dissolving 6.00 mL of 6000 ppm Taxol in 30.00 mL of distilled water. Two sample bottles were taken, and 2.00 g each of KPs was added to the sample bottles. 36.00 mL of 1000 ppm Taxol solution was added to one sample bottle (analyte), and 36.00 mL of distilled water was added to the other sample bottles (control). Both control and analyte were stirred at 200 rpm for 48 h. The products from the analyte were subsequently separated by centrifugation. Here, the filtrate and solid product were obtained from the analyte, while only a filtrate was obtained from the control. Next, a standard series of 300 ppm, 600 ppm, 900 ppm, and 1200 ppm Taxol solutions were prepared appropriately diluting the Taxol standard solution with distilled water. The same procedure was carried out for encapsulation in KP. The UV-Vis spectrometric studies at *λ*_max_ of 278 nm of the solutions were performed using distilled water as the reference.

Drug loading capacity (%) and encapsulation efficiency (%) were determined using the following equations:

(1) $$\begin{array}{c} \text{Encapsulation efficiency}=\frac{\text{total amount of Taxol}-\text{free Taxol}}{\text{total amount of Taxol}}\times 100\%, \end{array}$$

(2) $$\begin{array}{c} \text{Drug loading capacity}=\frac{\text{mass of entrapped drug Taxol }\left(\text{mg}\right)}{\text{keratin particle weight }\left(\text{g}\right)}\times 100\%. \end{array}$$

### 2.3. Determination of the Release Kinetics of Taxol Encapsulated in KPs

0.25 g of Taxol encapsulated in KPs was placed in cellulose dialysis tubes (cut-offs (MWCOs) of 12,000), which were placed, separately, in beakers containing 400.0 mL of buffer solutions of pH values of 5.0, 6.0, and 7.0. They were then placed on a thermostatic shaker maintained at 37°C and 100 rpm. 10.00 mL of the supernatant solution was withdrawn at 1 h time intervals for 8 and 24 hours while an equal volume of fresh buffer solutions was added. Taxol concentration of the buffer supernatant was determined using UV-Vis spectroscopy at *λ*_max_ = 278 nm using the buffer solution as the reference solution.

## 3. Results and Discussion

### 3.1. Characterization of Keratin Particles

**Figure 1:**
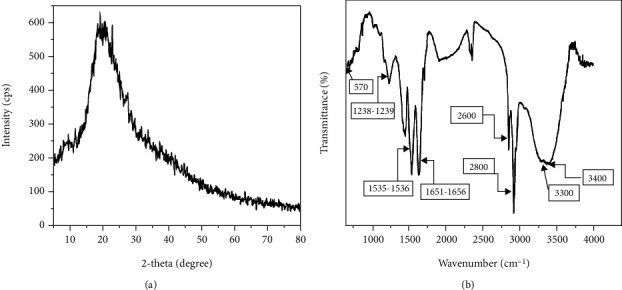
(a) XRD pattern obtained for keratin extracted from human hair. (b) FT-IR spectrum obtained for keratin from human hair.

The XRD shows no distinct peaks for any planes of the structure. Instead, it shows a broadband around 2*θ* angle 15°–25°, indicating that KPs consist of a semicrystalline- to amorphous-type structure (Figure 1(a)). This is in accordance with the typical structure of *α*-keratin where it has crystalline rod-like components made of right-handed alpha-helices, known as the intermediate filaments, embedded in an amorphous matrix (keratin-associated proteins) of approximately 18% of cystine as calculated from the overall amino acid composition and alanine, leucine, and arginine [36]. The crystalline intermediate filaments contribute to the XRD broadband since they are embedded in an amorphous outer layer.

The KPs prepared were also characterized using FT-IR spectroscopy, and the spectrum is shown in Figure 1(b). The FT-IR spectrum consists of a band centered around 3400 cm^−1^, which is due to O-H bond symmetric stretching vibration, 3300 cm^−1^ due to N-H bond asymmetric stretching, 2800 cm^−1^ due to C-H bond symmetric stretching in CH_3_ terminating groups, 2600 cm^−1^ due to C-H bond symmetric stretching in CH_2_ groups, in the narrow range 1651-1656 cm^−1^ due to C=O bond symmetric stretching, 1535-1536 cm^−1^ due to N-H secondary bond bending, 1238-1239 cm^−1^ due to C-N bond stretching, and 570 cm^−1^ due to C-S bond symmetric stretching. These bands are characteristic of those present in keratin, as shown in a previous study [37]. Therefore, it can be inferred that almost all the components in a protein molecule are present in this spectrum. The C=O and N-H bonds are very characteristic of a protein due to the presence of peptide linkages. In this case, the C-S bond becomes prominent due to cysteine, an essential amino acid in keratin proteins. Further, the normal long-chain hydrocarbon bonds (C-H) were observed due to side chains in many amino acids. The O-H broadband may be due to the presence of OH groups present in the protein structure, or it can be due to the fact that H_2_O vapor adsorbed onto KPs, which can take place due to its hydrophilic nature.

**Figure 2:**
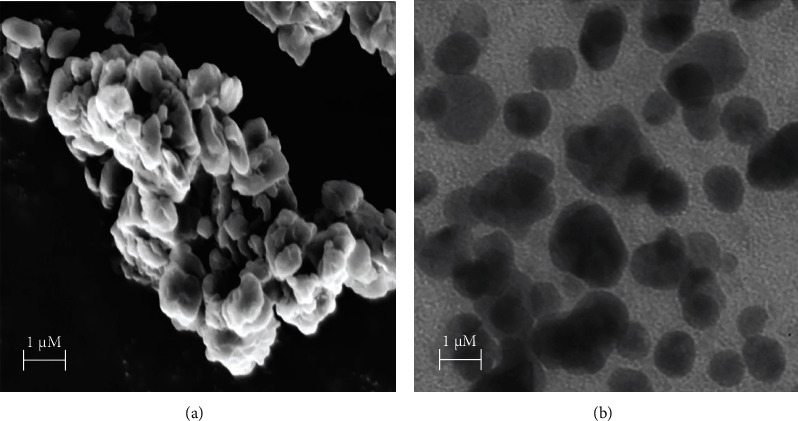
(a) TEM image and (b) SEM image of the extracted keratin particles.

Figure 2(a) shows the TEM image of the keratin microparticles synthesized before the encapsulation of the Taxol drug. The TEM images indicate that most of the particles were sized in the micrometer range. The microparticles have almost a spherical morphology. The conglomeration of microparticles was observed due to the interactions of the surface charges between the particles.  Figure 2(b) shows the SEM images of KPs. They are of 0.5-1.5 *μ*m average size comparable in size to those shown in the TEM image of isolated particles. Hence, KPs prepared in this work contain microparticles rather than nanoparticles. The morphology of the KPs shows a good porous nature as shown from the SEM image and is spherical as revealed from the TEM image.

Particle size distribution in the dispersed solution, measured using a laser light scattering-based Particle Size Analyzer, is shown in Fig. S3(a). The instrument plots the hydrodynamic radius of the colloidal particles in the suspension, represented in the *x*-axis. In contrast, the *y*-axis of the plot represents the parameter called Q3%, which represents the percentage of particles with a given hydrodynamic radius.

It can be inferred that the particle size of KPs varies in a wide range of 375 nm–1150 nm with an optimum value of 728.0 nm, above the 90% confidence level. It is not surprising to observe the absence of nanoscale particles because KNPs tend to aggregate together to form microparticles, which is a more stable size for KPs. The upper end of the size matches well with those observed in the TEM and SEM images. This suggests that smaller particles in the lower end of the 375 nm–1150 nm range readily agglomerate in the solid state, forming larger spherical particles.

The EDX report given in Fig. S3(b) clearly shows C, O, S, and Zn (with 79.02%, 20.25%, 0.71%, and 0.02% atomic percentages, respectively) which are the elements that are present in keratin. This also indicates that it does not consist of any adsorbed toxic species such as Cd or any other heavy metals, even in minute amounts, present in keratin extracted in this work.

### 3.2. Characterization Keratin Particles Treated with Taxol Solution

The SEM images obtained for KPs after treating with Taxol solution, as described in the previous section, are given in Fig. S4(a) and (b) with 25,000 and 50,000 magnifications, respectively. Particle size does not seem to have changed much due to this process indicating their stability at the micrometer scale. However, particles show a more open and flower-like arrangement when compared to those without Taxol treatment. The encapsulation of Taxol within KPs was studied by determining the encapsulation efficiency as detailed in the experimental section. The calibration curve was obtained using UV-visible spectroscopic determination of absorbance at *λ*_max_ of 278 nm [38]. The UV-visible absorption data for three repetitive measurements of the control sample containing 1000 ppm Taxol and the filtrate of the analyte sample after filtering off Taxol encapsulated in KPs were analyzed. The corresponding residual Taxol concentration in the filtrate of the analyte is 441 ppm. According to equation (1), it gives 56% encapsulation efficiency of Taxol in KPs. Furthermore, it clearly shows that a significant fraction of Taxol has been loaded (2.360 g g^−1^) within KPs (equation (2)).

### 3.3. Release Kinetics of Taxol from Keratin Particles

**Figure 3:**
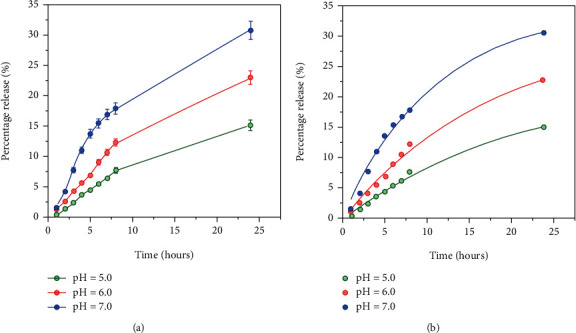
(a) Percentage release profiles of Taxol from those encapsulated in keratin particles in buffer solutions of different pH values. (b) Noyes-Whitney dissolution model-based fitting profile of Taxol release from KPs.

Profiles of Taxol release from KPs in buffer solutions at different pH values in vitro are shown in Figure 3(a). These profiles clearly show that Taxol release from KPs is a pH-dependent drug release profile. Most of the time, drug release profiles display a biphasic release trend, including an initial burst during the first few hours followed by a more sustained release in the following entire study duration [39, 40]. There was no initial burst type of release in our drug delivery system. It shows only a sustained, slow-release profile for a prolonged period, which signifies the strong encapsulation between Taxol in KPs. KPs are stable in mildly acidic pH conditions [41]. However, it is sensitive to neutral and basic pH conditions where KPs start to degrade. The drug release profiles of pH = 5.0, 6.0, and 7.0 clearly show that during the first 8 hours, which is crucial when it comes to anticancer drug delivery, the release of Taxol from KPs is around 15% at pH = 6.0 and even at pH = 7.0 (neutral pH), we can see that approximately 23% of total drug encapsulated in KPs is released after 8 hours. The pH of healthy tissues in the human body is about 6.8–7.3. It can be inferred that the release of drugs from keratin will not give harmful side effects compared to the drug administered in free form without encapsulation. Therefore, we can indicate that the side effects can be reduced up to a certain extent by encapsulation.

Further, during acidic pH values, the release of Taxol from KPs is slower. This is because KP is stable at low pH values. In general, cancerous cells contain a low pH value compared to a normal cell in the range of 6.4–7.0 [42]. This is due to abnormal glycolysis in cancer cells, which makes a high lactic acid to glucose ratio, making the interior of the cancer cells low in pH [43]. As such, it is expected that unless the Taxol encapsulated in KPs is selectively targeted only to cancer cells, a relatively higher amount of Taxol would be released at healthy cells than that at cancer cells. This can be achieved by attaching drug targeting ligands such as folic acid; a glutamate urea ligand known as DUPA; aminopeptidase N, also known as CD13; and FAP-*α*-targeting agents [40, 44]. Then, the obtained slow release can be used to reduce the dosage cycles given for cancer treatment, which is also a positive factor to reduce toxicity and side effects for the body by the drug. KPs can release the drug in a small amount within a longer period preventing toxicity risks. Hence, the drug delivery system can be considered acceptable for release during a prolonged period.

### 3.4. Application of Noyes-Whitney Diffusion Model and Mathematical Fitting Studies

The Noyes-Whitney dissolution model and a second-degree polynomial function were used to fit the drug release curves for Taxol release from KPs. Importantly, two different types of comparisons can be done to evaluate drug-releasing behavior. The theoretical predictions can either be compared with obtained independent experimental results, or the theory can be fitted to experimental data. The Noyes-Whitney equation was developed to characterize the process of solid dissolution [45, 46].

Dissolution is simply a process of dissociation or dispersion of a solute in a solvent, forming a chemically and physically stabilized, homogeneous molecular dispersion. The dissolution rate can be expressed as shown in

(3) $$\begin{array}{c} \frac{dm}{dt}=A\frac{D}{l}\left(C_{\text{s}}-C_{\text{b}}\right), \end{array}$$

where *dm*/*dt* is the solute dissolution rate (kg·s^−1^), *m* is the mass of dissolved material (kg), *t* is time (*t*), *A* represents the surface area of the solute particle (m^2^), *D* is the diffusion coefficient (m·s^−1^), *l* is the thickness of concentration gradient (m), *C*_s_ is the surface saturated concentration of solute particle (kg/mol·L^−1^), and *C*_*b*_ is the concentration in the bulk solvent/solution (kg/mol·L^−1^).

It is essential to rearrange the Noyes-Whitney fitting to solve the differential equation. Since the dissolution rate means the derivative of drug concentration over time, we can inverse it to get the relationship between the concentrations versus time:

(4) $$\begin{array}{c} \frac{dm}{dt}=A\frac{D}{l}\left(C_{\text{s}}-C_{\text{b}}\right). \end{array}$$

If we rearrange the Noyes-Whitney equation indicating the bulk concentration *C*_b_, where *V* represents the volume of the medium, we can get

(5) $$\begin{array}{c} \frac{dm}{dt}=\frac{{dC}_{\text{b}}}{dt}V,\\ \frac{{dC}_{\text{b}}}{dt}=A\frac{D}{lV}\left(C_{\text{s}}-C_{\text{b}}\right). \end{array}$$

Since *A*, *D*, *l*, and *V* are all constants, we find how the concentration will change through time by solving this differential equation. If we consider

(6) $$\begin{array}{c} \frac{{dC}_{\text{b}}}{dt}=Z\left(C_{\text{s}}-C_{\text{b}}\right),\text{where }Z=A\frac{D}{lV}, \end{array}$$

(7) $$\begin{array}{c} \frac{dC_{\text{b}}}{C_{\text{s}}-C_{\text{b}}}=Z\cdot dt. \end{array}$$

Solving this differential equation (7), we get −ln(*C*_s_ − *C*_b_) = *Z* · *t* + *k*, where *k* is a constant:

(8) $$\begin{array}{c} C_{\text{s}}-C_{\text{b}}=e^{\left(-\left(Z\cdot t+k\right)\right)},\\ C_{\text{b}}=C_{\text{s}}-e^{\left(-\left(Z\cdot t+k\right)\right)}. \end{array}$$

When *t* = 0, *C*_b_ = 0, *C*_s_ = *e*^−*k*^, where *k* = −lnC_s_, *C*_b_ = *C*_s_ − *e*^(−*Z* · *t*)^.*C*_s_ = *C*_s_(1 − *e*^(−*Z* · *t*)^):

(9) $$\begin{array}{c} C_{\text{b}}=C_{\text{s}}\left(1-e^{-\left(AD/Vl\right)t}\right). \end{array}$$

To get a relationship between total drug mass used (*m*^∗^) and the percentage drug release (*P*), equation (9) can be further transformed:

(10) $$\begin{array}{c} C_{\text{b}}=\frac{m}{V}=\frac{P\cdot m^{*}}{V}, \end{array}$$

(11) $$\begin{array}{c} P=\frac{V}{m^{*}}C_{\text{s}}\cdot \left(1-e^{-\left(AD/Vl\right)\cdot t}\right)\;\left(P\geq 0,P\leq 1,t\geq 0\right). \end{array}$$

If the Noyes-Whitney diffusion model is concerned, the diffusion coefficient (*D*) has an inversely proportional relationship between the viscosity of the solution, which can decrease the solute dissolution rate (*dm*/*dt*) with the increase of the viscosity of the solution. If the particle size decreases, the surface area of the particle is decreased, which makes *dm*/*dt* faster. Hence, the sizes of Taxol deposition masses play a role in changes in the solute dissolution rate. Apart from that, stirring of the solution and thermal agitations will decrease the diffusion gradient. Hence, the Taxol solute particles can be removed from the surface quickly to increase *dm*/*dt*. The other important thing is that when the pH solvent system is altered, the surface/saturation concentration *C*_s_ can also be changed accordingly. It depends on the solute (drug) characteristics and the solvent (cell plasma environment). Thus, the dissolution rate can be affected by the change of the *C*_s_ component. Cancer cells are normally at a low pH solvent environment, which affects the*C*_s_ component. We consider the characteristics of the Noyes-Whitney equation; with the increase of the time (*t*), the exponential part of equation ((10)) will be dropped down, and when the time goes to infinity, percentage drug release (*P*) will reach to a maximum upper limit (*P* = (*V*/*m*^∗^)*C*_s_).

Using the data in Figure 3(a), the Noyes-Whitney equation approach can interpret the original release curve we obtained where we can see that the release percentage of Taxol is gradually increasing. Still, the rate of the releasing rate is down within the first 10 hours for pH 5.0, 6.0 and 7.0. After 24 hours, the release is not fully finished, but the same trend of decreasing the release rate is maintained. The percentage drug release has a certain maximum value as proved in the Noyes-Whitney equation; surface/saturation concentration *C*_s_ of the drug plays an important role in determining whether the release percentage becomes completed or not.

#### 3.4.1. Experimental Data Fitting to Noyes-Whitney Model

If *m*^∗^/*V* ≥ *C*_s_ and time goes to infinity, the maximum upper limit of percentage drug release *P* = *V*/*m*^∗^*C*_s_ ≥ 1 and the drug is completely released from the matrix. But if this requirement is not fulfilled, the drug release will continue until it reaches the maximum release. In our system, even after 24 h percentage release has shown 15%, 23%, and 31% for pH = 5.0, 6.0, and 7.0 media, respectively, it still has not reached its maximum release. Had we done the release studies for more time, we would have ended up with the upper limit of percentage release if all other parameters are validated. This is essential and better for an effective drug releasing system with sustained-release action since slow release for a long period can kill cancer cells for *a* prolong period continuously. As Figures 3(a) and 3(b) show, the experimental release profiles, at each pH, match well with the theoretically fitted curves using the Noyes-Whitney model with *R*^2^ values of 0.9977, 0.9954, and 0.9902 for pH = 5.0, 6.0, and 7.0, respectively. Therefore, the drug release at neutral and near-neutral and slightly acidic pH values is mainly governed by the slow dissolution of the keratin carrier. The dissolution becomes faster as the pH is increased. It is possible that the drug release at pH 5.0 is mainly due to diffusion since the release kinetics is slow and the carrier 3-D structure is reasonably stable. It has been shown that the keratin structure is stable and that there is no break of covalent bonds or formation of new cross-links of the keratin summing up at low pH values [47]. The spiral structure restricts at low pH under 6.0 but does not disrupt predominantly into the random coil structure between pH 6.0 and 7.0 [48]. As such, the drug release in this pH range is primarily due to diffusion. At pH 7.0, the peptides PepD, PepE, PepF, PepG, and KP also present a negative net charge suggesting those peptides since the isoelectric point of *α*-keratin is below pH 7.0 [49]. Therefore, the ability to form H-bonds decreases at pH 7.0, thus making the structure more open and soluble. This is what we see for faster drug release at pH 7.0 compared to that at mildly acidic pH values. As such, *α*-keratin presents a good candidate for slow-release anticancer drug formulations, and together with cancer-targeting ligands, it can also act as a TDDS.

## 4. Conclusion

Toxin-free keratin microparticles isolated from human hair were used as carriers in the targeted delivery of Taxol to cancerous cells. The formulation gives acceptable release kinetics at the mildly acidic pH conditions prevailing in cancer cells without the carrier degradation. This formulation is expected to reduce Taxol cytotoxicity to healthy cells and slowly release the drug in a constant amount for a prolonged period only at the vicinity of the cancer cells, thus destroying them selectively. We have also used the Noyes-Whitney dissolution model to fit the drug release process, and the approach is successful and can well explain the experimental data we obtained. Observed drug release kinetics has also been described using the chemical structure of the carrier and its stability at different pH values.

## Data Availability

Data supporting the results can be requested by anyone from the corresponding author if needed.

## Supplementary Materials

Supplementary Materials Supplementary 01: introduction to structure and function of keratin. Supplementary 02: introduction to anticancer drug Paclitaxel (Taxol). Fig. S2: chemical structure of Paclitaxel (Taxol). Supplementary 03: Fig. S3: SEM images of keratin particles treated with Taxol solution: (a) 25,000 magnification; (b) 50,000 magnification. Supplementary 04: Fig. S4: (a) particle size distribution in the solution phase of keratin measured by laser light scattering and (b) the elemental composition of keratin as measured by TEM-EDAX.
