# Supplementary material for: Noyes-Whitney Dissolution Model-Based pH-Sensitive Slow Release of Paclitaxel (Taxol) from Human Hair-Derived Keratin Microparticle Carriers
Source: Biomed Res Int. 2021 May 7;2021:6657482. doi: 10.1155/2021/6657482 (PMC8128610; doi:10.1155/2021/6657482)
Supplement: Supplementary Materials — Supplementary 01: introduction to structure and function of keratin. Supplementary 02: introduction to anticancer drug Paclitaxel (Taxol). Fig. S2: chemical structure of Paclitaxel (Taxol). Supplementary 03: Fig. S3: SEM images of keratin particles treated with Taxol solution: (a) 25,000 magnification; (b) 50,000 magnification. Supplementary 04: Fig. S4: (a) particle size distribution in the solution phase of keratin measured by laser light scattering and (b) the elemental composition of keratin as measured by TEM-EDAX. [file 6657482.f1.docx]

**Supporting information**

Noyes-Whitney dissolution model-based pH-sensitive slow release of paclitaxel (Taxol) from human hair-derived keratin microparticle carriers

Wimalasiri V.W ^1^, Dunuweera S.P ^1, 2^, Dunuweera A.N ^3^, Rajapakse R.M.G ^1, 2^

Corresponding author: Rajapakse R.M.G: [rmgr@pdn.ac.lk](mailto:rmgr@pdn.ac.lk)

^1^Department of Chemistry, Faculty of Science, University of Peradeniya, Sri Lanka

^2^Postgraduate Institute of Science, University of Peradeniya, Sri Lanka

^3^Department of Basic Science, Faculty of Allied Health Sciences, University of Peradeniya, Peradeniya 20400, Sri Lanka

**Table of contents**

**S1** Introduction to structure and function of Keratin

**S2**: Introduction to anticancer drug Paclitaxel (Taxol)

**Fig. S2**: Chemical structure of Paclitaxel (Taxol)

**Fig.S3**: SEM images of keratin particles treated with Taxol solution. (a)25,000 magnification (b)50,000 magnification

**Fig.S4**: (a) Particle size distribution in the solution phase of keratin measured by LASER light scattering and (b) the elemental composition of keratin as measured by TEM-EDAX.

**S1:**

Hair essentially consists of keratin, a fibrous structural protein which is a member of the superfamily of intermediate filament proteins, describes a form of crystalline rod-like components made of right-handed alpha-helices (the intermediate filaments, IFs) embedded in an amorphous matrix (keratin associated proteins) that have a relatively high amount of cystine (and references therein), 18% as calculated from the overall amino acid composition and alanine, leucine and arginine as well coiled coil structure is formed by twisting the two polypeptide chains together in left-handed direction. These dimers utilize the many cysteine amino acids found in α-keratins and bonded together with disulfide bonds. Then these dimers arrange align way with their termini ends with the termini ends of other dimers and two of these new chains bond length-wisely and form protofilament. Then protofibrils are formed and from four of these protofibrils make the intermediate filament (IF) which is the basic subunit of the alpha keratin. This IF structures are finally embedded in keratin matrix which is either high in cysteine or glycine, tyrosine, and phenylalanine residues. The different alignments, types and matrices of IF s leads to form large variation in α-keratin structures found in mammals.

Between the exogenous proteins and the hair keratin, numerous bonds such as ionic, hydrophobic and hydrogen bonds are formed. Further, covalent binding, such as that in disulphide bonds, may also occur to form the coiled structure.

Keratin, a major hair protein, is able to create stronger or weaker interactions with other hair proteins. For example, the presence of disulphide bonds in cysteine residues of the hair keratin protein determine the maintenance of the shape of the hair and can be altered by various chemical teatments. The disulphide bonds are much stronger, with the closer arrangement of thiol groups and make these bonds easier to form, and in consequence the hair is much curlier. Hair fibre is sensitive to changes in the pH values. When in contact of alkaline reducing agents with the cortex, the disulphide bonds break and rearrangement can be happened as well as the stretching the spiral coil of keratin molecules. In a practical pH range, peptides and amino acids have high ability to diffuse into the hair fibre cortex, high substantively and the ability to aid recovery from hair cuticle damage. Due to pH value plays a major role for the activity of proteins and significantly influences the denaturation of soluble proteins, proteins exhibit their active properties within certain ranges of values of pH. Alkaline pH is favorable to cystine disruption and to the hydrolysis of the amide bonds and formation of lanthionine and lysinoalanine.

According to the amino acid composition of hair does not differ significantly, before and after exposure to strong acid (low pH value) indicating that the acidic treatment does not hydrolyze the protein chain. There is no any break of covalent bonds or formation of new cross-links of the keratin summing up, the treatment with low pH solution does not change significantly the tensile strength of the keratin material. At pH 7.0, the peptides PepD, PepE, PepF, PepG and KP also present a negative net charge suggesting those peptides will not favor the adsorption mechanism onto the hair fibre. The coil structure restricts at low pH under 6.0 but not disrupts predominantly into the random coil structure between pH 6.0-7.0. But a random coil structure can be produced between that pH range (6.0-7.0) with having high mobility assuming that the coiled coil structure preferentially disrupts between pH 6.0-7.0. Further the proteins begin the rapid hydrolysis at pH 9.2. In the case of alkalis, the hydrolysis of keratin occurs irreversibly above pH 9.2 with great destructive effect. But some of the coiled-coil structure still remain even at pH 9.6 which is the pH of the strongest permanent waving lotion.

**S2:** Taxol is a plant-based taxane alkaloid, isolated from the bark of the Pacific yew tree, *Taxus brevifolia*, which is used particularly in the treatment of breast, ovarian, lung, bladder, prostate, melanoma, esophageal, as well as other types of solid tumor cancers. It has also been used in Kaposi's sarcoma, since 1993. The main action of Taxol has been the ability to halt cell division. Usually, these drugs damage the RNA or DNA that gives the signal to copy itself in cell division. If the cells are unable to divide, they then die. Taxol also has the ability to induce cell suicide (self-death or apoptosis). Common side effects occurring in more than 30% of patients under Taxol treatment include low blood counts, hair loss, arthralgias and myalgias, pain in the joints and muscles which are usually temporary occurring 2 to 3 days after Taxol and resolve within a few days. Keratin is the carrier used to encapsulate Taxol and is a fibrous structural protein present in hair, hoofs, nails, horn, wool, feathers, and of the epithelial cells. In the epithelium, keratin serves important functions such as protection and some keratins have been found to regulate important cellular activities such as protein synthesis and cell growth. Hair consist of 95% keratin, which is a fibrous, helicoidal protein (shaped like a helix) which is insoluble in water, thereby ensuring impermeability and protection. Hair keratin is a α-keratin considering its secondary structure and is rich in cysteine which is mainly responsible for formation of disulfide bonds between molecules, adding rigidity to the entire structure.


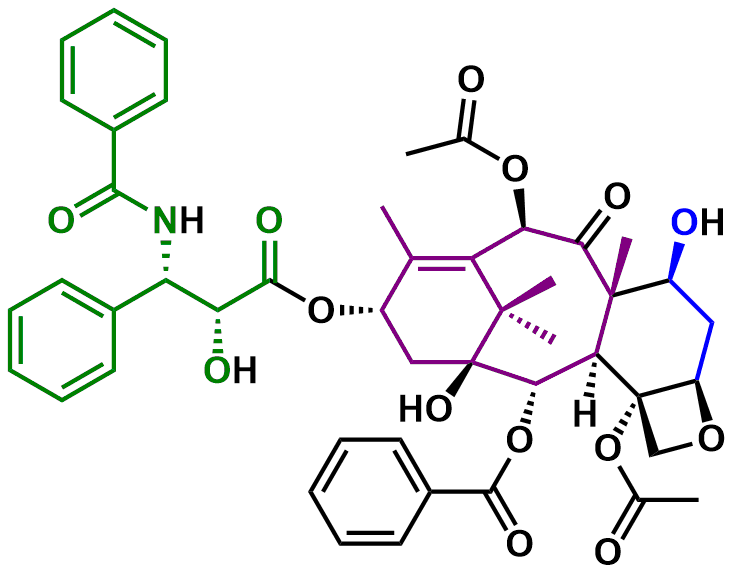


#### Fig. S2: Chemical structure of Paclitaxel (Taxol)

#### File: Taxol Full2.png - Wikimedia Commons. (2016). Retrieved 21 September 2020, from <https://commons.wikimedia.org/wiki/File:Taxol_Full2.png>

**S3:**

(b)

(a)


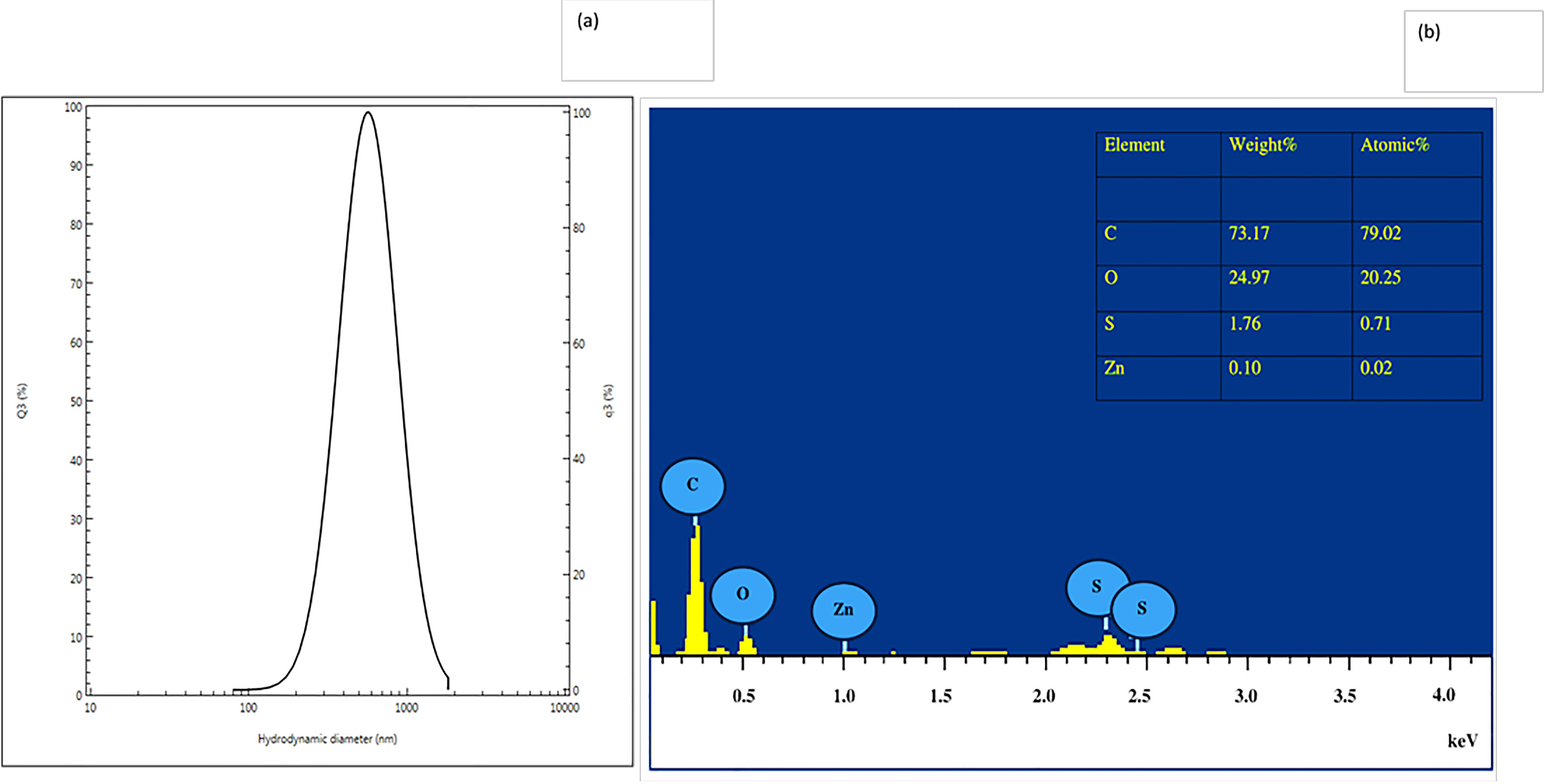

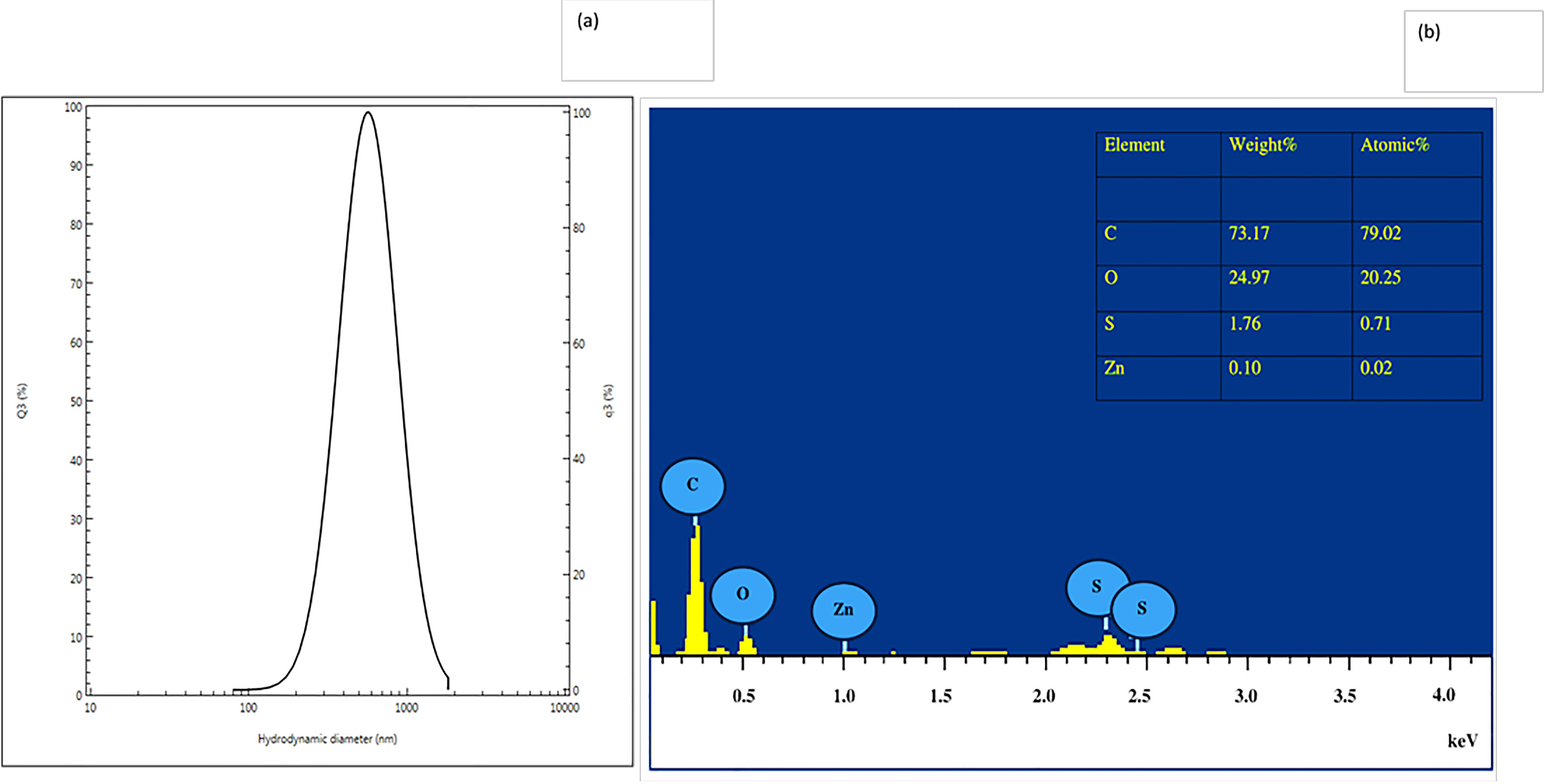


Fig.S3: SEM images of keratin particles treated with Taxol solution. (a)25,000 magnification (b)50,000 magnification

**S4:**

(b)

(a)


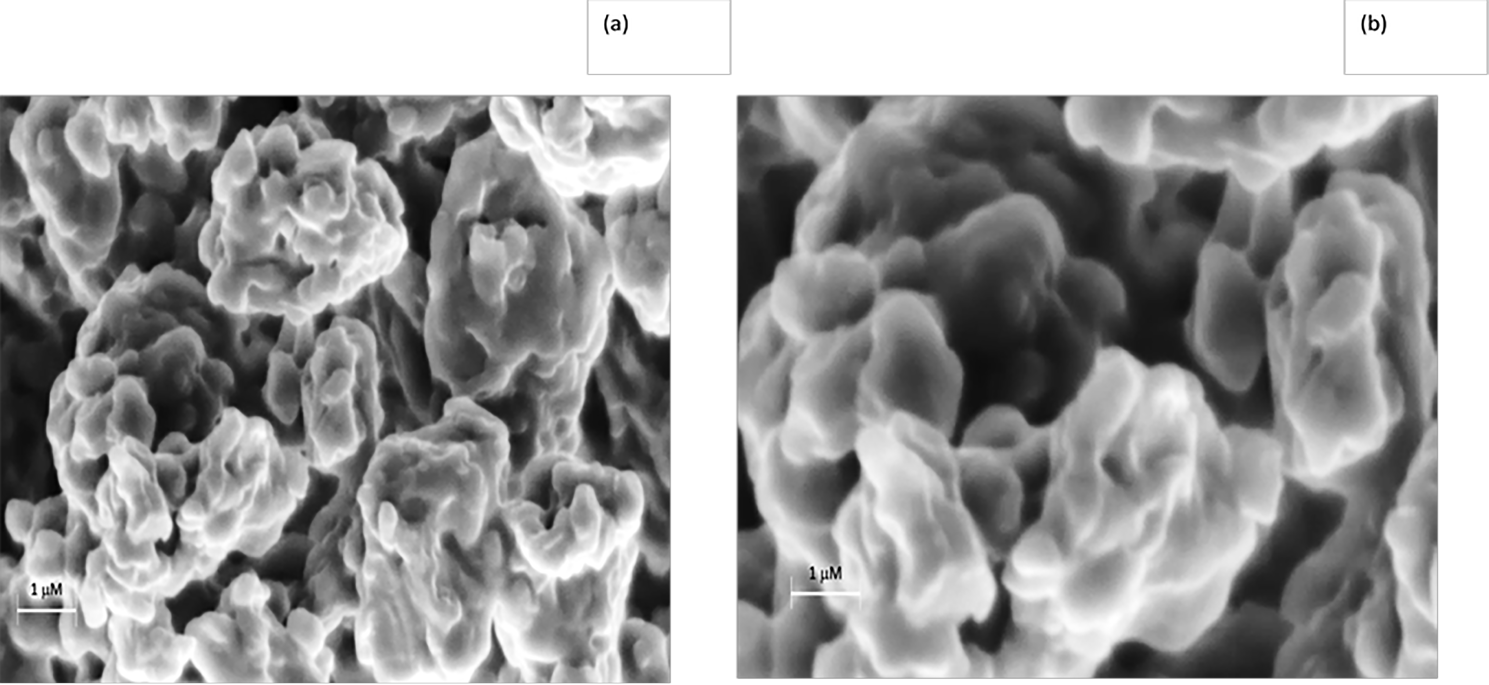

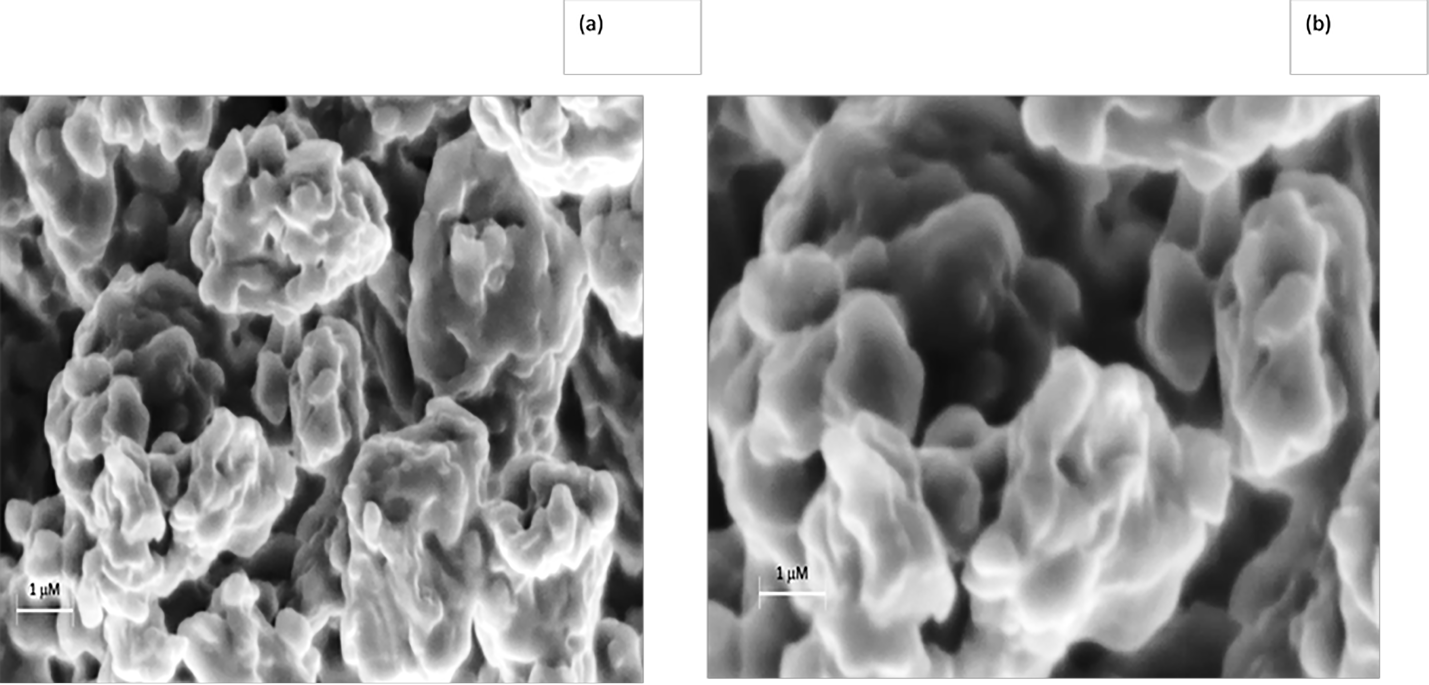


Fig.S4: (a) Particle size distribution in the solution phase of keratin measured by LASER light scattering and (b) the elemental composition of keratin as measured by TEM-EDAX
